# Image Features of Magnetic Resonance Angiography under Deep Learning in Exploring the Effect of Comprehensive Rehabilitation Nursing on the Neurological Function Recovery of Patients with Acute Stroke

**DOI:** 10.1155/2021/1197728

**Published:** 2021-09-10

**Authors:** Rui Yang, Ying Zhang, Miao Xu, Jing Ma

**Affiliations:** Intensive Care Unit, Critical Care Medicine, Affiliated Hongqi Hospital of Mudanjiang Medical University, Mudanjiang 157011, Heilongjiang, China

## Abstract

This study was to explore the effects of imaging characteristics of magnetic resonance angiography (MRA) based on deep learning on the comprehensive rehabilitation nursing on the neurological recovery of patients with acute stroke. In this study, 84 patients with acute stroke who were treated in hospital were selected as the research objects, and they were rolled into a control group (routine care) and an experimental group (comprehensive rehabilitation care). The dense dilated block-convolution neural network (DD-CNN) algorithm under deep learning for cerebrovascular was adopted to assess the effect of comprehensive rehabilitation care on the neurological recovery of patients with acute stroke. The results showed that the Berg scale scores, Fugl-Meyer scores, and *Functional Independence Measure* (FIM) scores of the experimental group of patients after 6 weeks and 12 weeks of comprehensive rehabilitation nursing were greatly different from those before treatment, showing statistical differences (*P* < 0.05). Compared with conventional magnetic resonance imaging (MRI) images, MRA images based on CNN algorithm, Dense Net algorithm, and DD-CNN algorithm can more clearly show the patient's cerebral artery occlusion. The average dice similarity coefficient (DSC) values of CNN algorithm, Dense Net algorithm, and DD-CNN algorithm were determined to be 84.3%, 95.7%, and 97.8%, respectively; the average sensitivity (Sen) values of the three algorithms were 76.1%, 95.4%, and 96.8%, respectively; and the average accuracy (Acc) values were 87.9%, 96.3%, and 97.9%, respectively. Thus, there were statistically obvious differences among the three algorithms in terms of average values of DSC, Sen, and Acc (*P* < 0.05). The MRA images processed by the DD-CNN algorithm showed that the degree of neurological recovery of the experimental group was observably greater than that of the control group, and the difference was statistically obvious (*P* < 0.05). In short, the image features of MRA based on the deep learning DD-CNN algorithm showed good application value in studying the effect of comprehensive rehabilitation nursing on the neurological recovery of patients with acute stroke, and it was worthy of promotion.

## 1. Introduction

Stroke is an acute cerebrovascular disease with high morbidity, disability, mortality, and recurrence [1]. Among them, acute ischemic stroke patients have rapid changes in their condition, high risk, and high mortality. The treatment effect is extremely time-dependent. The treatment of the disease has always emphasized early diagnosis, early treatment, early recovery, and early prevention of recurrence [2, 3]. The symptoms of acute stroke vary widely. When the patient suddenly experiences weakness or numbness in one limb (with or without face), numbness or slanted mouth angle on one side, slurred speech or difficulty in understanding language, eyes gaze to one side, loss or blurred vision in one or both eyes, dizziness accompanied by vomiting, severe headaches, vomiting, disturbance of consciousness or convulsions, and other symptoms, the possibility of stroke should be considered [4, 5]. At present, the commonly used imaging examination methods for stroke mainly include computed tomography (CT) examination and magnetic resonance imaging (MRI) examination of the patient's brain parenchyma to check for cerebral infarction, cerebral hemorrhage or brain tumor, and brain inflammation [6, 7]. Computed tomography angiography (CTA), intravascular ultrasound (IVUS), extracorporeal vascular ultrasound (VU), or magnetic resonance angiography (MRA) are used for patient vascular examination to confirm whether the blood vessels are blocked, ruptured, bleeding, and other pressure [8]. If the above examination still fails to confirm the diagnosis, the digital subtraction angiography (DSA) is feasible, and the stroke function examination also includes CT perfusion imaging and magnetic resonance perfusion imaging [9, 10]. In recent years, MRA technology has gradually become the preferred method of angiography, in which technology realizes vascular imaging by comparing the difference in magnetization between static tissue and flowing blood and the strength of the spin signal. Its advantages are no radiation, low trauma, clear imaging of tissue background and blood vessels, and short imaging time [11].

With the continuous development of deep learning in the field of medical image segmentation, more and more disease image diagnosis and efficacy evaluation have used deep learning related algorithm content. In recent years, deep learning cerebrovascular segmentation algorithms have emerged in an endless stream, which is optimized continually based on the convolution neural network (CNN) [12, 13]. For example, the currently emerged algorithms based on regional similarity model, active contour-based model algorithms, clustering analysis algorithms, algorithm based on Maximum a Posteriori Probability (MAP), and algorithms based on Markov random field (MRF) are optimized and updated based on the traditional CNN algorithms.

In addition, due to the rapid onset of acute stroke and local neurological function defects, early treatment and rehabilitation care are needed. At present, the rehabilitation care methods for various stroke sequelae all over the world include community rehabilitation nursing, systematic rehabilitation nursing, and comprehensive rehabilitation nursing [14, 15]. Studies have shown that the recovery period of stroke patients' limb dysfunction and language dysfunction is more than five years, and effective functional training can realize the reorganization of the structure and function of the central nervous system at the brain injury site and realize the plasticity recovery of the brain [16]. Based on this, this study hoped to use MRA imaging detection methods to explore the effect of comprehensive rehabilitation nursing on the neurological recovery of patients with acute stroke. A DD-CNN algorithm for MRA image features of stroke patients was hereby designed. It was hoped that the algorithm quality evaluation and the comprehensive rehabilitation nursing effect evaluation for acute stroke can be used to verify the value of proposed algorithm and comprehensive rehabilitation nursing in the diagnosis and treatment of patients with clinical acute stroke.

## 2. Materials and Methods

### 2.1. Research Objects

Eighty-four acute stroke patients who were admitted to the hospital from October 2019 to September 2020 were selected as the research objects, including 51 males and 33 females, aged 47–78 years old (with an average age of 63.4 ± 4.7 years old). All patients were randomly divided into an experimental group and a control group, with 42 people in each group. Patients in the experimental group received comprehensive rehabilitation nursing, and patients in the control group received conventional nursing. MRA imaging scans were performed before and after treatment. The study had been approved by the ethics committee of the hospital, and the patients and their families included in the study were known and signed the informed consents.

The inclusion criteria were defined as follows: patients who were diagnosed by MRI of the brain, and patients who met the diagnostic criteria established by the *Fourth National Cerebrovascular Academic Conference* in 1995; patients with sequelae of stroke who showed dysfunction such as limb hemiplegia, and the course of the disease >6 months; patient whose condition was stable after medical treatment; and patients who and their family had signed an informed consent form. During the treatment, the family and the patient had a high degree of cooperation and adhered to the treatment.

The exclusion criteria were defined as follows: patients with primary stroke with asymptomatic cerebral infarction; stroke patients with a course of less than 6 months; stroke patients with no sequelae of stroke; patients with hemiplegia of the limbs or other dysfunctions caused by other encephalopathy (such as brain tumor, brain injury, and brain parasitic disease); stroke patients who had re-stroke or severe complications; patients with sequelae of stroke with severe language dysfunction and behavior disorder; patients with other neurological diseases affecting the cognitive function; patients with medical history of various malignant tumors; and patients with severe mood disorders.

### 2.2. Rehabilitation Nursing Methods of Two Groups of Patients

The patients in the control group took routine nursing, which included basic treatment and nursing and activities of daily living (ADL) training once a day. ADL training run through the patient's daily life, allowing the patient to gradually restore the ability to live independently. On the basis of the nursing method of the control group, patients in the experimental group received comprehensive rehabilitation nursing intervention once a day. The comprehensive rehabilitation nursing intervention treatment items included relaxation training, palm microvibration therapy, sports rehabilitation training, and psychological counseling. The relaxation training process required targeted relaxation training for the patient's bones, muscles, and joints with breathing, and the time was about 15 minutes. The palm microvibration therapy was a treatment method that applied vibration to the parts and muscle groups that produce joint contractures to achieve a soothing and relaxing effect on the corresponding parts. The average vibration frequency was 100∼200 times/min, and the duration of each part was about 1 minute. In the exercise rehabilitation training of the experimental group of patients, the balance ball was used. The training content included knee flexion and extension exercises, swing exercises with foot joints as fulcrums, waist hip and abdominal oblique muscle twisting and stretching training, and knee Joint flexion and extension exercises. The daily duration was about 20∼30 minutes. In the process of psychological counseling of patients, humorous stories, timely and effective emotional support to patients and their families, and certain incentive mechanisms can be used to praise patients for their progress in the treatment process, so as to enhance patients' self-confidence, enhance their self-nursing ability, and master more effective self-management skills.

### 2.3. Data Collection and Comprehensive Rehabilitation Effect Evaluation Indicators of Patients

Basic information of the patients was collected, including gender, age, course of disease, and nature of the disease. The Berg Balance Scale (BBS), Fugl-Meyer motor assessment scale (FMA), and Functional Independence Measure (FIM) were performed before and after treatment to evaluate the patient's recovery condition. The Berg Balance Scale is one of the main scales for assessing the balance ability of stroke patients, covering 14 items. The minimum score for each item is 0 points, and the maximum is 4 points, so the total score is 56 points; the higher the BBS score, the better the balance function. FMA can evaluate the limb function of patients with hemiplegia very well. Each action of the scale is divided into three grades of 0–2 points, with a full score of 100 points, including 66 points for upper limbs and 34 points for lower limbs. The higher the FMA score, the better the motor function. FIM includes two categories: cognitive function and physical movement, with a total of 18 items. The highest FIM score is 126 points, of which the motor function score is 91 points, and the cognitive function score is 35 points; the lowest score is 18 points. If FIM = 126 points, it means that the patient is completely independent; if FIM = 108∼125 points, it means that the patient is basically independent; if FIM = 90∼107 points, it means that the patient is conditionally independent or extremely lightly dependent; if FIM = 72∼89 points, the patient is mildly dependent; if FIM = 54∼71 points, the patient is moderately dependent; if FIM = 36∼53 points, the patient is heavily dependent; if FIM = 19∼35 points, it indicates that the patient is extremely dependent; if FIM = 18 points, it suggests that the patient is completely dependent [17].

### 2.4. The Architecture of DD-CNN Algorithm Based on Deep Learning

**Figure 1:**
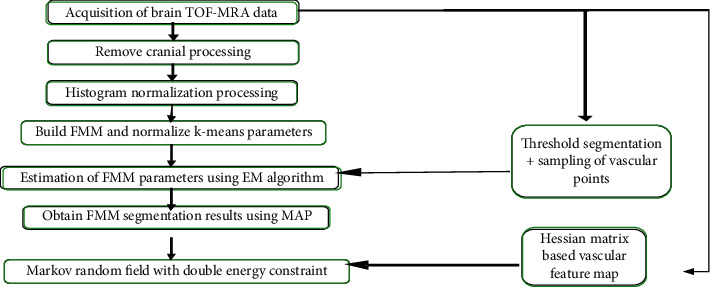
The overall frame diagram of device-independent FMM-MRF.

Since the existing cerebrovascular segmentation models mostly use finite mixture model (FMM) to model the original data, there are many shortcomings such as parameter differences across devices and low accuracy in cerebrovascular segmentation; and Markov random field (MRF) multimodal field system can further improve the progress of cerebrovascular extraction [18]. Therefore, the FMM and MRF system are combined to construct a FMM-MRF cerebrovascular segmentation algorithm that is not limited to equipment in this study. The overall process of the algorithm included data preprocessing, FMM establishment, and EM algorithm based on blood vessel knowledge for FMM parameter estimation, and MRF optimization process based on dual energy constraints. The overall results of the model are shown in Figure 1.

**Figure 2:**
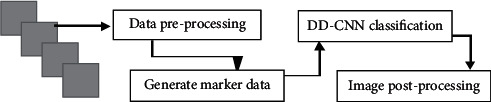
The processing flowchart of DD-CNN.

On this basis, the DD-CNN adapted to the characteristics of blood vessels was introduced. The network was roughly composed of four parts: data preprocessing, labeling data generation, DD-CNN classification, and postprocessing. The specific flowchart is shown in Figure 2.

When DD-CNN processing was performed, the Hessian matrix-based filtering and enhancement of blood vessel preprocessing technology were introduced in this study to reduce the unevenness of the self-selected signal intensity in the blood caused by the blood laminar flow in the MRA image. Firstly, *N* (*z*) was set to the gray value at the point *z*=[*z*_1_,…, *z*_*m*_], and *σ* represented the scale, then the corresponding matrix *G*_*a*,*b*_(*α*, *z*) at the point *z* in the image can be expressed as

(1) $$\begin{array}{c} G_{a,b}\left(\sigma ,z\right)=\sigma^{2}N\left(z\right)*\frac{\partial^{2}}{\partial z_{a}\partial z_{b}}F\left(\sigma ,z\right). \end{array}$$

In the above equation, *a*, *b*=1,…, *m*; ^*∗*^ represented the convolution operation, and *F*(*σ*, *z*) was an *N*-order Gaussian function, which could be expressed as follows:

(2) $$\begin{array}{c} F\left(\sigma ,z\right)={\left(2\pi \sigma^{2}\right)}^{-\left(N/2\right)}\exp\left(\frac{-zz^{T}}{2\sigma^{2}}\right). \end{array}$$

The feature value of each *z* point can be expressed as eig{*H*(*σ*, *Z*)}⟶*λ*_*a*_, *a*=1,…, *m*. The feature values were sorted according to the absolute value of the eigenvalue, so that they could satisfy *|λ*_*a*_*|* ≤ *|λ*_*a*+1_*|*, and then the feature value of the corresponding Hessian matrix satisfied the following relationship:

(3) $$\begin{array}{c} |\lambda_{1}|\ll |\lambda_{2}|\cap \lambda_{2}\approx \lambda_{3}. \end{array}$$

Then, according to the tubular shape of normal blood vessels, the blood vessel response function *T* was defined as

(4) $$\begin{array}{c} T=|\left(\lambda_{2}-\lambda_{1}\right)\lambda_{2}\lambda_{3}|\left[\frac{3}{|2\lambda_{2}-\lambda_{1}|+|\lambda_{3}|}\right]. \end{array}$$

To improve the low-contrast situation of blood vessels in MRA images, it can redefine the *λ*_3_ under the filter scale *σ* to obtain

(5) $$\begin{array}{c} \lambda_{e}\left(\sigma \right)=\left\{\begin{array}{cc} \lambda_{3}, & \lambda_{3}>\varepsilon \max_{z}\left\{\lambda_{3}\left(z,\sigma \right)\right\},\\ \varepsilon \max_{z}\left\{\lambda_{3}\left(z,\sigma \right)\right\}, & 0<\lambda_{3}>\varepsilon \max_{z}\left\{\lambda_{3}\left(z,\sigma \right)\right\},\\ 0, & \text{otherwise.} \end{array}\right. \end{array}$$

To effectively enhance the vascular structure of the elliptical cross-section, the vascular response function was changed to equation (6) by defining *λ*_*e*_⟶(*λ*_*e*_ − *λ*_2_) in equation (5):

(6) $$\begin{array}{c} T=\left\{\begin{array}{cc} 0, & \lambda_{3}\geq 0\cup \lambda_{2}\geq 0,\\ 1, & \lambda_{2}<\lambda_{3}/2<0,\\ |\lambda_{2}^{2}\left(\lambda_{e}-\lambda_{2}\right)|{\left[\frac{3}{|\lambda_{2}|+|\lambda_{3}|}\right]}^{3}, & \text{otherwise.} \end{array}\right. \end{array}$$

Then, according to FMM, the intracranial proportion of the cerebrovascular was estimated, so as to determine that there was a threshold *Q* that can define the enhancement result, and the threshold *Q* satisfied equations (7) and (8):

(7) $$\begin{array}{c} W=\frac{\sum_{p=1}^{S}\mu \left(f_{p},Q\right)}{S}, \end{array}$$

(8) $$\begin{array}{c} \mu \left(f_{p},Q\right)=\left\{\begin{array}{cc} 1, & f_{p}\geq Q\\ 0, & f_{p}<Q \end{array}\right.. \end{array}$$

In the above equations, *f*_*p*_ was the enhancement value of multiscale blood vessels in the brain tissue area, *S* represented the total number of voxels in the brain tissue area, and *W* referred to the intracranial volume rate of cerebral blood vessels estimated by FMM. Next, the following transformations could be performed on the results of multiscale vessel enhancement:

(9) $$\begin{array}{c} ℑ=\frac{1}{1+{\left(Q/T\right)}^{2}}. \end{array}$$

In equation (9), *ℑ* referred to the converted result of the blood vessel feature map, and its numerical value represented the probability value of the point being a blood vessel voxel.

### 2.5. Cerebrovascular Segmentation Indicators in MRA Image of Patients with Acute Stroke

In this study, dice similarity coefficient (DSC), positive predictive value (PPV), sensitivity (Sen), and accuracy (Acc) were used to evaluate the cerebral vascular segmentation in patients with acute stroke. The specific calculation equations were given as follows:

(10) $$\begin{array}{c} \text{DSC}=\frac{2A}{2A+C+D},\\ \text{Sen}=\frac{A}{A+D},\\ \text{PPV}=\frac{A}{A+B},\\ \text{Acc}=\frac{A+C}{A+B+C+D}. \end{array}$$

In the four equations above, *A*, *B*, *C*, and *D* represented the number of true positive, the number of false positive, the number of true negative, and the number of false negative results, respectively.

### 2.6. Statistical Analysis

The test data were processed using SPSS19.0 statistical software, the measurement data was expressed in the form of mean ± standard deviation ($\bar{x}\pm s$), and the comparison of the mean between groups was performed by *t* test. The count data was expressed by percentage (%), and the *χ*^2^ test was used. *P* < 0.05 indicated that the difference was statistically significant.

## 3. Results

### 3.1. General Information of the Two Groups of Patients

**Figure 3:**
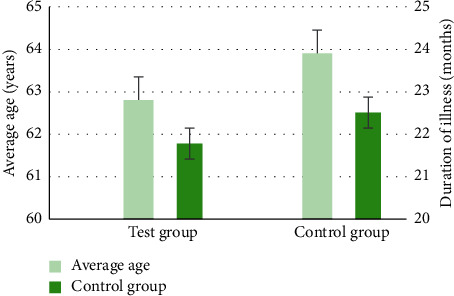
Comparison of the average age and the course of disease between the two groups of patients.

**Figure 4:**
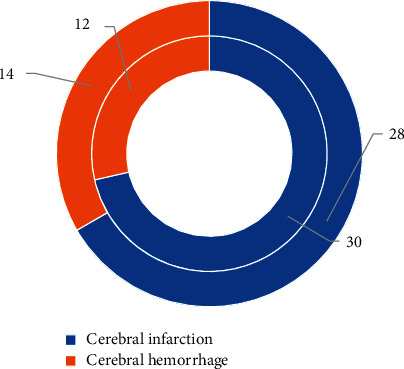
Comparison on nature of the disease for patients in the two groups.

Figure 3 shows the comparison of the average age and the course of disease between the two groups of patients, and Figure 4 shows the nature of the disease for patients in the two groups. Figure 3 illustrates that the average ages of the experimental group and the control group were 62.8 ± 5.1 years old and 63.9 ± 4.3 years old, respectively, and the age difference between the two groups was not statistically significant (*P* > 0.05); and the average course of disease of patients in the experimental group was 21.78 ± 6.27 months, in which that in the control group was 22.51 ± 4.36 months, showing no statistically great difference (*P* > 0.05). As shown in Figure 4, in the two groups of patients, 30 acute stroke patients in the experimental group had cerebral infarction, and 12 cases had cerebral hemorrhage; and there were 28 cases of cerebral infarction and 14 cases of cerebral hemorrhage in the control groups, so there was no statistically significant difference in the number of different types of lesions between the two groups (*P* > 0.05).

### 3.2. Treatment Effect Evaluation of Comprehensive Rehabilitation Nursing for Two Groups of Patients

**Figure 5:**
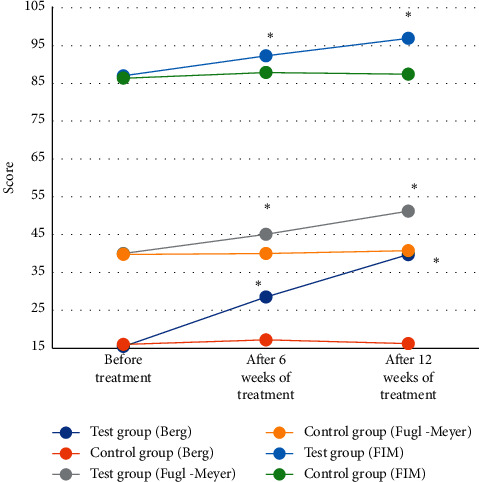
Effect evaluation of comprehensive rehabilitation nursing for two groups of patients. ^*∗*^The difference was statistically obvious in contrast to the value before treatment (*P* < 0.05).

Figure 5 shows the evaluation of the comprehensive rehabilitation nursing effect of the two groups of patients. The Berg scale scores, Fugl-Meyer scores, and FIM scores before and after the treatment of the two groups of patients were compared. The Berg scale scores, Fugl-Meyer scores, and FIM scores of the experimental group patients after 6 weeks after comprehensive rehabilitation nursing treatment were 15.45 points, 40.05 points, and 87.01 points, respectively; while those in the control group were 16.01 points, 39.78 points, and 86.35 points, respectively. The Berg scale scores, Fugl-Meyer scores, and FIM scores of the experimental group patients after 12 weeks after comprehensive rehabilitation nursing treatment were 39.76 points, 51.23 points, and 96.89 points, respectively; while those in the control group were 16.23 points, 40.78 points, and 87.44 points, respectively. Therefore, it was concluded that the three scores of the experimental group were obviously improved compared to the scores before treatment, and the differences were statistically significant (*P* < 0.05), while the Berg scale score, Fugl-Meyer score, and FIM score of the control group before and after routine nursing were not greatly different, showing no statistical differences (*P* > 0.05).

### 3.3. MRA Image Changes Based on Deep Learning Algorithm

**Figure 6:**
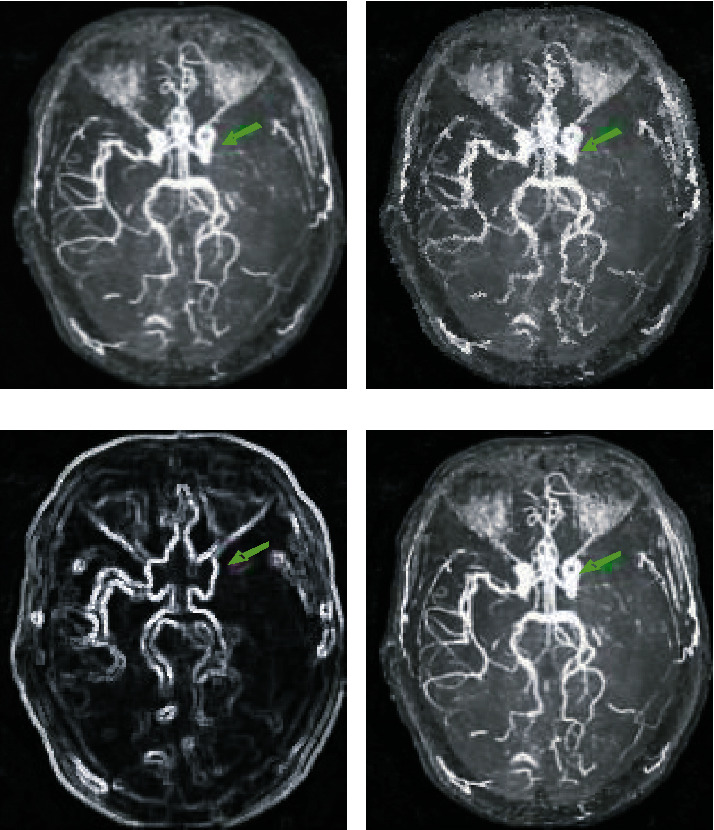
MRA image of a 55-year-old male patient. (a) the original MRA image; (b) the MRA image processed by the CNN algorithm; (c) the MRA image processed by the dense net algorithm; and (d) the MRA image processed by the DD-CNN algorithm.

Figure 6 shows the patient's MRA images under different algorithm processing conditions, and it was a 75-year-old male patient with acute stroke. Compared with conventional MRI images, MRA images based on CNN algorithm, Dense Net algorithm, and DD-CNN algorithm were clearer, and the artery occlusion in the left brain of the acute stroke patient was clearer, and the imaging was more detailed.

### 3.4. Quantitative Evaluation Results of MRA Images of Different Algorithms

**Figure 7:**
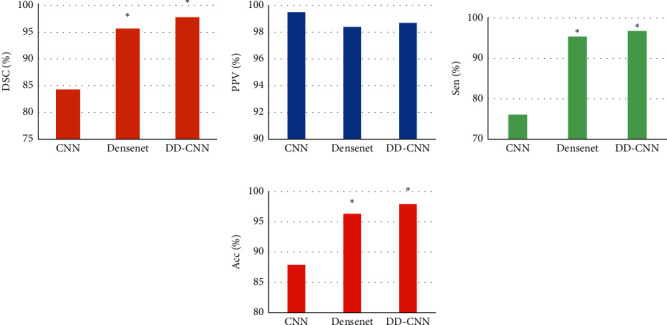
Quantitative evaluation results of MRA images of different algorithms. ^*∗*^The difference was statistically obvious in contrast to the value before treatment (*P* < 0.05).

The effects on MRA images of different algorithms were quantitatively evaluated, and the results are given in Figure 7. As illustrated, the average DSC values of the CNN algorithm, Dense Net algorithm, and DD-CNN algorithm were 84.3%, 95.7%, and 97.8%, respectively. Among them, the average DSC value of the CNN algorithm was obviously different from the other two algorithms, showing statistical significance (*P* < 0.05). The average values of PPV of NN algorithm, Dense Net algorithm, and DD-CNN algorithm were 99.5%, 98.4%, and 98.7%, respectively, so the differences in the average values of PPV of the three algorithms were not statistically significant (*P* > 0.05). The average Sen values of CNN algorithm, Dense Net algorithm, and DD-CNN algorithm were 76.1%, 95.4%, and 96.8%, respectively, and the average Sen value of the CNN algorithm was remarkably different from that of the other two algorithms, which was statistically significant (*P* < 0.05). The average Acc values of NN algorithm, Dense Net algorithm, and DD-CNN algorithm were 87.9%, 96.3%, and 97.9%, respectively, so that the CNN algorithm was much lower in contrast to the Acc values of the other two algorithms, with statistical differences (*P* < 0.05).

### 3.5. Analysis on MRA Images of Patients with Acute Stroke Treated with Comprehensive Rehabilitation Nursing to Evaluation of the Neurological Function Recovery

**Figure 8:**
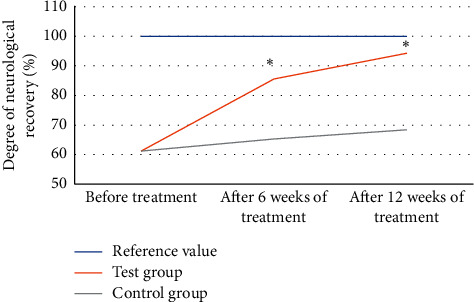
Analysis of MRA images of patients with acute stroke treated with comprehensive rehabilitation nursing to evaluation of the neurological function recovery. ^*∗*^The difference was statistically obvious in contrast to the value before treatment (*P* < 0.05).

The MRA images of patients with acute stroke treated with comprehensive rehabilitation nursing were analyzed to the evaluation of the neurological function recovery, and the results are illustrated in Figure 8. As revealed, before the nursing treatment, there was no observable difference in the neurological function assessment results of the two groups of patients, which were all 61.2% (*P* < 0.05); after 6 weeks and 12 weeks of different nursing treatments, MRA images processed based on the DD-CNN algorithm showed that the neurological function recovery of the experimental group was 85.6% and 94.3%, respectively, which were remarkably improved than those of the control group (65.3% and 68.4%, respectively), and the difference was statistically obvious (*P* < 0.05).

## 4. Discussion

Due to a large number of related researches on rehabilitation nursing after stroke in recent years, it is believed that timely and effective postoperative rehabilitation nursing treatment can effectively improve the patient's balance ability, motor function, walking ability, and ability of daily living [18, 19]. In addition, it can alleviate the psychological pressure of stroke patients to a certain extent, enhance the self-confidence of patients during prognosis rehabilitation through the encouragement in nursing treatment, and improve the degree of cooperation in rehabilitation treatment [20]. In addition, the current application of imaging technology in the clinical application of disease diagnosis and recovery judgment of acute stroke patients has been relatively common. Various artificial intelligence algorithms designed for the misdiagnosis caused by the traditional use of the subjective judgment of physicians have been obtained. And the results were good [21]. Therefore, a DD-CNN algorithm under deep learning for MRA image features of stroke patients was designed in this study, hoping that the MRA image features based on the deep learning DD-CNN algorithm can be used to evaluate the performance of acute stroke patients before and after comprehensive rehabilitation care, so as to verify the application value of this algorithm and the comprehensive rehabilitation nursing treatment in the recovery of neurological function of clinical stroke.

The results of this study showed that the Berg scale scores, Fugl-Meyer scores, and FIM scores for patients in the experimental group who were treated with comprehensive rehabilitation nursing for 6 weeks and 12 weeks were greatly different from those before treatment, with statistical significance (*P* < 0.05). There was no obvious difference in the Berg scale score, Fugl-Meyer score, and FIM score of the control group before and after routine nursing, which was not statistically significant (*P* > 0.05). It can be concluded that comprehensive rehabilitation nursing can effectively improve the balance ability, upper and lower limb motor function, and ability of daily living of patients with acute stroke, effectively improving the quality of life of patients with prognosis. Such results were similar to the research results of Malavé et al. [22], which confirm that comprehensive rehabilitation nursing can effectively improve the ability of daily living of patients with acute stroke and help patients restore balance and motor functions. At the same time, compared with conventional MRI images, MRA images based on CNN algorithm, Dense Net algorithm, and DD-CNN algorithm are clearer, which can improve the receptive field area of MRA images and speed up the processing speed of the MRI image of the region of interest localization stage and the fine segmentation stage, so that the imaging of the middle cerebral artery occlusion of acute stroke patients can be more detailed, and the focus of the disease can be accurately located. The quantitative evaluation results of the MRA impact of different algorithms show that the average DSC, Sen, and Acc of CNN algorithm were significantly different from those of the other two algorithms, showing statistical significance (*P* < 0.05). The average PPV of the three algorithms were 99.5%, 98.4%, and 98.7%, respectively, and the difference in the average PPV of the three algorithms was not remarkable and was not statistical (*P* > 0.05). It shows that the MRA processed by the DD-CNN algorithm based on deep learning can better improve the DSC, Sen, and Acc of the segmentation result compared with the traditional CNN algorithm and the MRA image segmentation effect of the Dense Net algorithm. The study of Qi et al. also used similar algorithm evaluation criteria, and the results showed that end-to-end deep learning technology reconstructed nine times undersampled free-breathing whole-heart coronary MRA (CMRA) images in nonrigid motion correction (MoCo), and it showed good denoising effect, faster reconstruction time, and better blood vessel visual image clarity [23]. In addition, before the nursing treatment, there was no significant difference between the conventional MRA image and the MRA image processed by the DD-CN N algorithm in the neurological function assessment results of the two groups of patients. After 6 weeks of different nursing treatments, the MRA images processed based on the DD-CNN algorithm showed that the neurological recovery of the experimental group was visibly greater than that of the control group, showing statistical differences (*P* < 0.05).

## 5. Conclusion

A deep learning DD-CNN algorithm was designed for image features of MRA for cerebral blood vessels of stroke patients in this study and applied to verify the effect of comprehensive rehabilitation nursing on the neurological function recovery of patients with acute stroke. The results proved that the algorithm showed better accuracy and sensitivity of MRA image segmentation than the traditional CNN algorithm and Dense Net algorithm and verified that the comprehensive rehabilitation therapy can effectively recover the neurological function of patients with acute stroke. However, the number of selected patient samples in this study was small, and the source of which was single, so that it only discussed the different manifestations of stroke patients at different lesions in more detail, and it was impossible to verify the impact of these features on the accuracy of prediction. In the future, it will consider increasing the sample size of patients and further adopt the analysis method of multicenter cooperation for research. All in all, the results of this study could provide a good theoretical basis for the application of MRA images based on the deep learning DD-CNN algorithm in the clinical diagnosis of acute stroke patients.

## Data Availability

The data used to support the findings of this study are available from the corresponding author upon request.
